# Machine Learning Models for Early Autism Spectrum Disorder Detection: Systematic Review With a Pediatric and Engineering Perspective

**DOI:** 10.7759/cureus.115572

**Published:** 2026-09-01

**Authors:** Chrisphine Onyango, Lispah N Wanjiku, Calvince Oketch

**Affiliations:** 1 Behavior Services, Anderson Centre for Autism, Staatsburg, USA; 2 Children Residential Services, Anderson Center for Autism, Staatsburg, USA

**Keywords:** artificial intelligence, autism screening, autism spectrum disorder, clinical translation, deep learning, early detection, explainable ai, machine learning, pediatric diagnosis, prediction models

## Abstract

Autism spectrum disorder (ASD) is a heterogeneous neurodevelopmental condition for which earlier identification may facilitate timely developmental assessment and intervention. This systematic review synthesized evidence on machine learning (ML)/ deep learning (DL) approaches for early ASD detection, with particular attention to pediatric populations, data modalities, model architectures, reported diagnostic performance, validation strategies, methodological quality, and clinical and engineering translation. PubMed and IEEE Xplore were searched through July 29, 2026. The supplied database exports contained 794 PubMed records and 455 IEEE Xplore records. After removal of seven records published after the prespecified cutoff, 1242 records remained. Thirty-eight duplicates were removed, leaving 1204 unique records for screening. Following title/abstract screening, 314 reports underwent detailed record-level eligibility assessment, of which 237 were classified as record-level eligible on the basis of available titles, abstracts, bibliographic metadata, and accessible record information. The identified evidence encompassed questionnaire and clinical-information models, home-video and behavioral analysis, speech and acoustic features, eye tracking, EEG/MEG, MRI/fMRI, wearable sensors, and multimodal approaches. Reported diagnostic performance varied substantially across populations, modalities, algorithms, and validation settings. Some retrospective studies reported very high classification accuracy, whereas independent and cross-cultural validation generally produced more variable performance. For example, an ML model based on medical and background information reported an AUROC of 0.895 during development and 0.790 in independent validation, while a real-world evaluation of an AI-based diagnostic system reported sensitivity of 99.1% and specificity of 81.6% among determinate outputs. Because the available evidence was heterogeneous and full-text reports were not available for all candidate studies, quantitative pooling and definitive study-level risk-of-bias assessment were not considered defensible. PROBAST+AI domains were therefore assessed only when sufficient methodological information was available, with otherwise unclear or not-assessable judgments. Across the evidence base, external validation, prevention of data leakage, dataset shift, explainability, privacy, fairness, reproducibility, computational requirements, and clinical workflow integration remain major barriers to translation. ML and DL approaches show promise as adjunctive screening and risk-classification technologies, but the available evidence does not support replacing specialist ASD assessment with autonomous AI diagnosis. Future research should prioritize prospective multicenter validation, transparent reporting, independent testing, clinically meaningful explainability, privacy-preserving methods, health-equity evaluation, calibration, and deployment within real-world pediatric clinical workflows.

## Introduction and background

Autism spectrum disorder and the need for earlier identification

Autism spectrum disorder (ASD) is a heterogeneous neurodevelopmental condition characterized by persistent differences in social communication and interaction together with restricted or repetitive patterns of behavior [1-4]. Clinical presentation varies substantially across individuals, complicating early recognition and increasing the need for accessible and reliable assessment pathways. Surveillance and global prevalence studies demonstrate the substantial public-health burden of ASD and the importance of timely identification and access to services [5,6].

Early identification is clinically important because developmental interventions are generally most useful when access to assessment and support is not unnecessarily delayed [7-10]. However, comprehensive ASD diagnosis relies on detailed clinical history, structured or semi-structured behavioral assessment, and specialist judgment. These processes require trained clinicians and may be difficult to access in some settings [2-4]. This has motivated interest in scalable adjunctive approaches that could help identify children who should receive further specialist evaluation.

Artificial intelligence in ASD detection

Artificial intelligence (AI), particularly machine learning (ML) and deep learning (DL), can model nonlinear relationships in high-dimensional data and has been applied to behavioral questionnaires, video, neuroimaging, and other data sources relevant to ASD [11-22]. Commonly applied models include support vector machines, random forests, gradient-boosting methods, and deep neural networks, which identify patterns within these data to support ASD classification and early detection. Behavioral studies have used instruments such as the Autism Diagnostic Interview-Revised (ADI-R), Autism Diagnostic Observation Schedule (ADOS), Social Responsiveness Scale (SRS), and Mobile Autism Risk Assessment (MARA), often applying feature-selection methods to reduce the number of items required for classification [11-16].

Video-based approaches have examined whether short home recordings can support remote screening [23-26]. Neuroimaging studies have used resting-state functional MRI (rs-fMRI), autoencoders, support vector machines, recurrent neural networks, and other learning approaches to evaluate functional-connectivity patterns [27-33].

More recent work has expanded beyond these established modalities. The PubMed evidence includes investigations involving EEG and functional near-infrared spectroscopy (fNIRS), eye tracking, home video, medical and developmental information, and multimodal behavioral assessment. For example, a 2026 systematic review identified 27 peer-reviewed EEG/fNIRS studies published between 2019 and 2024 and reported substantial heterogeneity in preprocessing workflows, probe montages, and classification models. Similarly, a 2023 systematic review and meta-analysis identified 24 studies involving 1396 individuals using eye-tracking data for ASD classification, demonstrating that eye-tracking ML research had already developed into a substantial evidence area [34]. Figure 1 illustrates the artificial intelligence framework for early autism detection.

**Figure 1:**
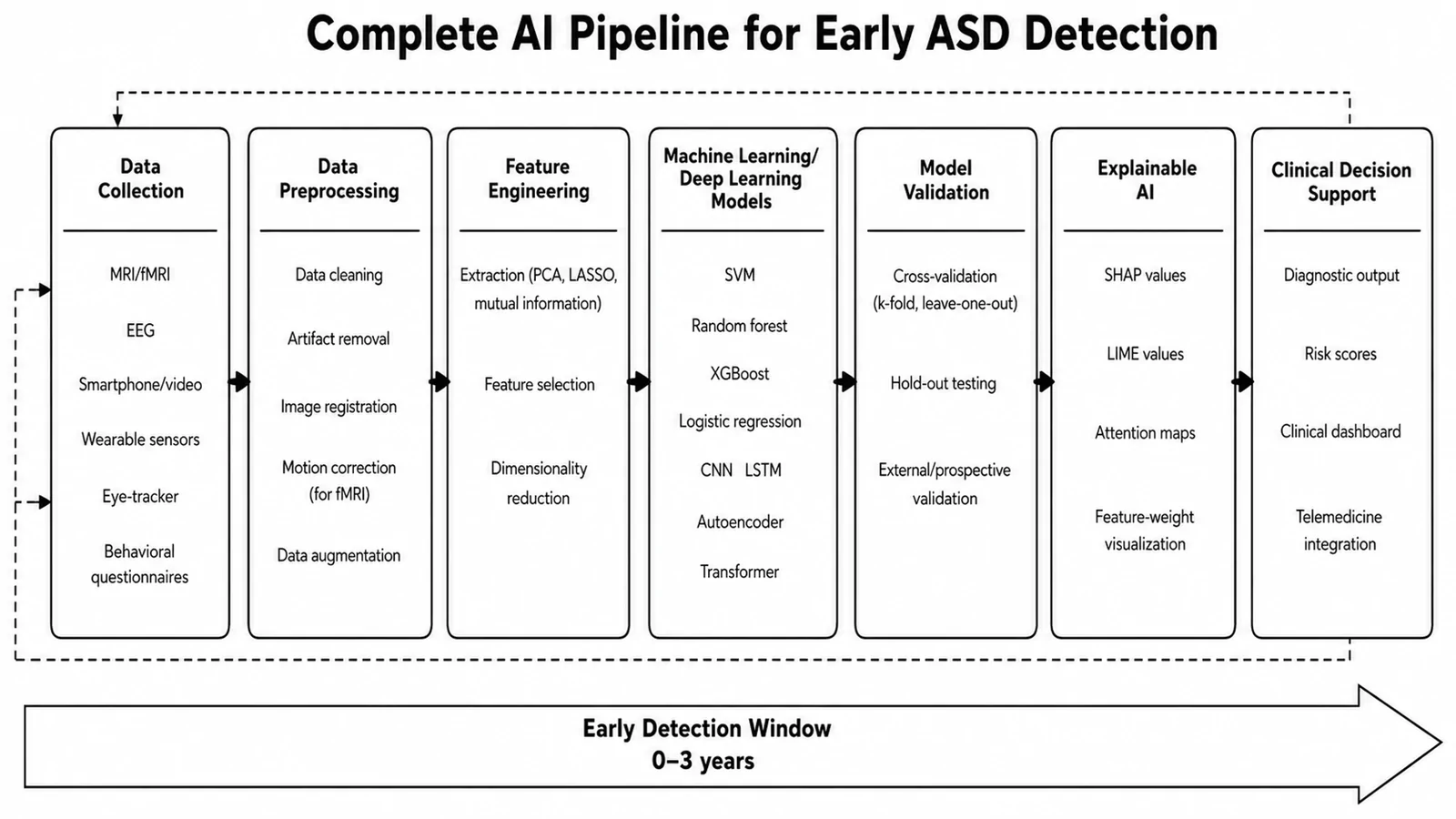
Artificial intelligence framework for early autism detection Conceptual framework illustrating the progression from data collection and preprocessing through feature engineering, machine learning/deep learning modeling, model validation, explainable AI, and clinical decision support for early autism detection. The figure was created by the authors using Python (Matplotlib). fMRA - functional magnetic resonance imaging; PCA - principal component analysis; SVM - support vector machine; CNN - convolutional neural network; LSTM - long short-term memory; LIME - local interpretable model-agnostic explanations

Review objectives

Previous reviews have addressed behavioral ML, MRI-based classification, eye-tracking systems, and broader AI applications in ASD [21,22,34-36]. The present review extends this literature by considering multiple data modalities together and by explicitly examining engineering and deployment-related factors.

The objectives were to 1) systematically identify and summarize ML/DL methods relevant to early ASD detection with a pediatric focus; 2) summarize reported diagnostic performance and determine whether quantitative pooling is justified; 3) characterize validation strategies and methodological limitations; 4) assess engineering readiness, including external validation, explainability, reproducibility, computational requirements, privacy, and clinical deployment considerations; and 5) identify priorities for future research and translation.

## Review

Study design and reporting

This study was conducted as a systematic review with structured evidence synthesis and an engineering assessment of ML/DL approaches relevant to ASD detection. Reporting was guided by Preferred Reporting Items for Systematic reviews and Meta-Analyses (PRISMA) 2020 [37]. Quantitative pooling was planned only when studies were sufficiently comparable in population, diagnostic target, outcome definition, and validation design. Because the evidence was heterogeneous, results were synthesized primarily descriptively. The review was not prospectively registered in the International Prospective Register of Systematic Reviews (PROSPERO) [38]. This is acknowledged as a limitation. Eligibility criteria were defined using a Population, Intervention/Index Method, Comparator, Outcome, and Study Design (PICOS)-informed framework [39]. The review focused on pediatric ASD detection, but some historically influential ML studies used mixed-age datasets. Mixed-age studies were retained only when the published report included pediatric participants and met the operational pediatric eligibility rule. Findings from such studies are explicitly interpreted as mixed-age rather than exclusively pediatric evidence.

Eligibility criteria

Eligibility was defined using a PICOS-informed framework. The review focused on pediatric ASD detection, but some historically influential ML studies used mixed-age datasets. Mixed-age studies were retained only when the published report included pediatric participants and met the operational pediatric eligibility rule. Findings from such studies are explicitly interpreted as mixed-age rather than exclusively pediatric evidence (Table 1).

**Table 1:** Inclusion and exclusion criteria for the systematic review ASD - autism spectrum disorder; AI - artificial intelligence; ML - machine learning

Domain	Inclusion criteria	Exclusion criteria
Population	Children <18 years, or mixed-age datasets meeting the operational pediatric eligibility rule	Adult-only studies; animal models
Index method	Machine-learning, deep-learning, or AI classification methods	Traditional statistical analyses without an ML/AI classification component
Outcome	ASD-related diagnostic, screening, detection, or classification outcome versus neurotypical or clinical comparator groups	Severity prediction, treatment response, skill acquisition, therapy outcome, or other outcomes without ASD classification
Study design	Peer-reviewed original research or full peer-reviewed conference proceedings	Reviews, meta-analyses, editorials, case reports, commentaries, and non-research publications
Language	English	Non-English publications

Information sources and search strategy

PubMed and IEEE Xplore were searched from inception through July 29, 2026. The PubMed strategy was the following: ("Autism Spectrum Disorder" OR "ASD" OR "Autism") AND ("Machine Learning" OR "Deep Learning" OR "Artificial Intelligence" OR "SVM" OR "CNN" OR "Neural Network") AND ("Child" OR "Pediatric" OR "Infant" OR "Toddler") AND ("Diagnosis" OR "Detection" OR "Screening" OR "Classification"). The IEEE Xplore strategy was the following: ("Autism" OR "ASD" OR "Autism Spectrum Disorder") AND ("Machine Learning" OR "Deep Learning" OR "Artificial Intelligence" OR "Neural Networks" OR "SVM" OR "CNN") AND ("Child" OR "Pediatric" OR "Infant" OR "Toddler") AND ("Diagnosis" OR "Detection" OR "Screening" OR "Classification")

The supplied PubMed export contained 794 records, while the IEEE Xplore export contained 455 records. Three PubMed records and four IEEE Xplore records were dated after the July 29, 2026 cutoff and were removed (Table 2).

**Table 2:** Database search yield after application of the July 29, 2026, publication cutoff

Database	Records in supplied export	Records removed after cutoff	Records retained
PubMed	794	3	791
IEEE Xplore	455	4	451
Total	1249	7	1242

Study selection

In accordance with PRISMA 2020 terminology, a record refers to the title or abstract, or both, of a report indexed in a database or website; a report refers to a document supplying information about a particular study; and a study refers to the underlying investigation, which may be represented by one or more reports. These terms are used distinctly throughout this review.

The exports yielded 1249 records before date filtering and 1242 records after application of the July 29, 2026 cutoff. Thirty-eight duplicate records were removed, leaving 1204 unique records. The 1204 records were screened against the eligibility criteria. Eight hundred ninety records were excluded during initial title/abstract screening. The remaining 314 reports underwent detailed record-level eligibility assessment (Table 3, Figure 2).

**Table 3:** Record-level study-selection summary

Selection stage	Records/reports
PubMed records retained after date filtering	791
IEEE Xplore records retained after date filtering	451
Total records after date filtering	1242
Duplicates removed	38
Unique records screened	1204
Records excluded during initial screening	890
Reports retained for detailed record-level assessment	314
Reports excluded during detailed assessment	77
Record-level eligible reports	237
Primary studies included in detailed study-level synthesis	20

**Figure 2:**
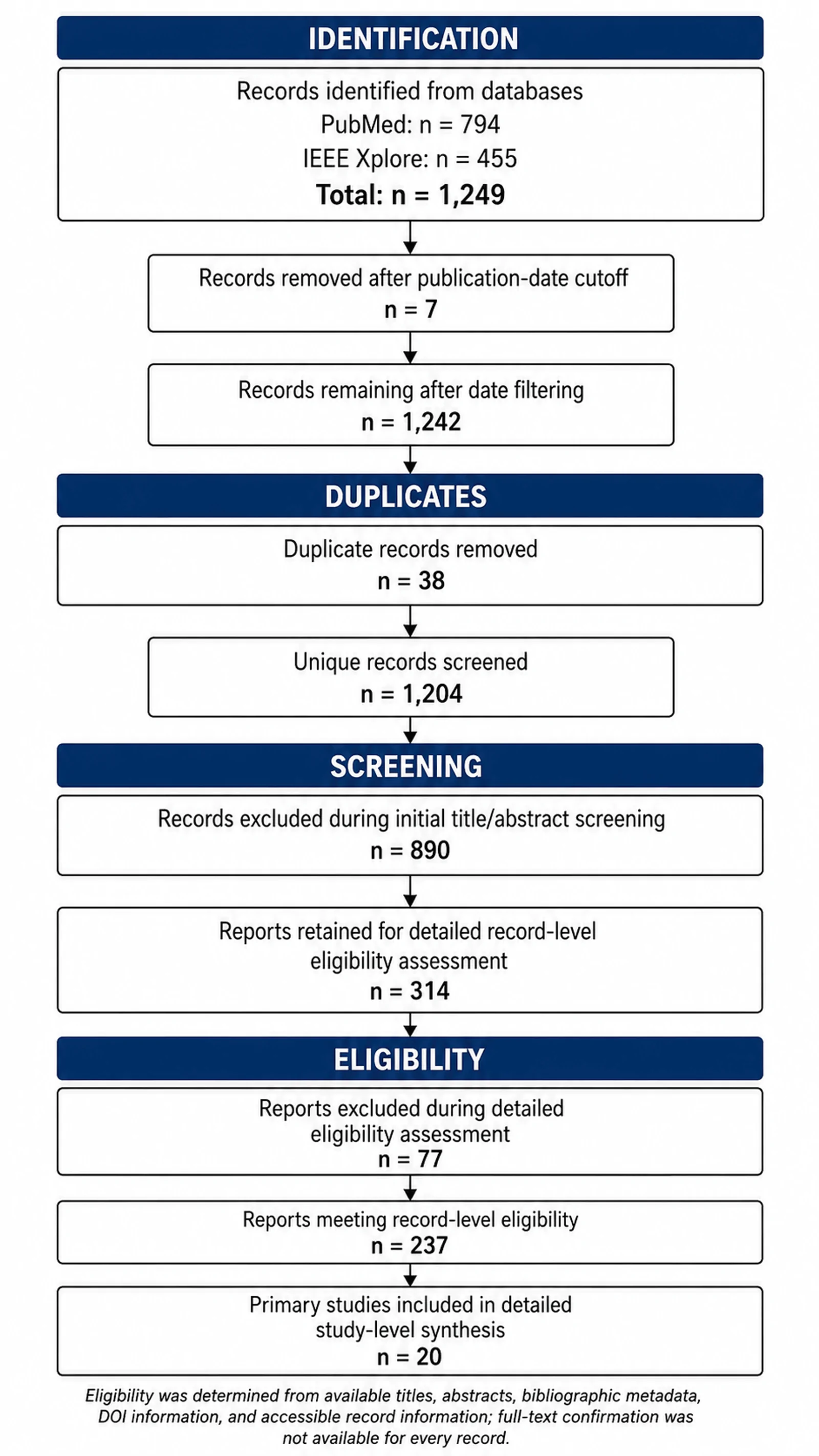
PRISMA 2020 flow diagram of the study-selection process The diagram summarizes database identification, publication-date filtering, duplicate removal, initial screening, and detailed record-level eligibility assessment. PRISMA - Preferred Reporting Items for Systematic Reviews and Meta-Analyses

Because complete full-text reports were not available for all candidate reports, eligibility assessment for those records was based on the available titles, abstracts, bibliographic metadata, DOI information, and other accessible record information. A total of 237 reports met the prespecified eligibility criteria at the record level and constituted the record-level eligible evidence base. Of these, 20 primary studies for which sufficient study-level information was available were included in the detailed study-level synthesis. Selection for the detailed synthesis was based on the availability of sufficient study-level information to extract and characterize key methodological and outcome variables required for the planned synthesis. The remaining record-level eligible reports contributed to characterization of the broader evidence landscape but were not treated as full-text-confirmed included studies.

Data extraction

A standardized extraction framework was used to collect bibliographic information, population characteristics, sample size and age range, ASD and comparator definitions, data modality, model architecture, feature-selection methods, validation strategy, and diagnostic performance metrics. Additional information was extracted on computational requirements, explainability, code availability, privacy considerations, and deployment considerations. Where information was not available in the abstract or record, it was coded as not reported (NR) rather than inferred. The extracted information was independently reviewed by a second author for accuracy and consistency, with any discrepancies resolved through discussion among the authors.

Risk of bias and reporting assessment

Reporting considerations for prediction-model studies were also informed by the Transparent Reporting of a multivariable prediction model for Individual Prognosis Or Diagnosis (TRIPOD) framework [40].

Prediction Model Risk of Bias Assessment Tool (PROBAST) [41] provided the earlier prediction-model risk-of-bias framework, while PROBAST+AI [42] was used as the preferred framework for models involving regression or artificial-intelligence methods (Table 4). The four principal assessment domains are participants/data sources, predictors, outcome, and analysis. Because many newly identified records were available only as abstracts, methodological details such as leakage prevention, missing-data handling, feature-selection timing, participant-level data separation, calibration, and external validation were frequently unavailable. Therefore, no unsupported low- or high-risk judgment was assigned when the available information was insufficient.

**Table 4:** Abstract-level PROBAST+AI assessment framework PROBAST+AI - Prediction Model Risk of Bias Assessment Tool + Artificial Intelligence; ASD - autism spectrum disorder

Domain	Abstract-level assessability	Principal information required
Participants/data sources	Frequently unclear	Recruitment, exclusions, representativeness, sample-size adequacy
Predictors	Partially assessable	Predictor definition, preprocessing, feature selection
Outcome	Usually more assessable	ASD reference standard and outcome definition
Analysis	Frequently unclear	Missing data, leakage, tuning, validation, calibration
Overall risk of bias	Not assigned when evidence is insufficient	Requires sufficient information across domains

Statistical analysis

Quantitative pooling was considered only when at least two studies reported sufficiently comparable outcomes for the same diagnostic task and validation setting. The evidence base remained heterogeneous in populations, modalities, datasets, outcome definitions, model architectures, and validation methods. Consequently, no pooled AUC, pooled diagnostic odds ratio, summary ROC curve, or pooled sensitivity/specificity estimate was considered defensible. Diagnostic metrics were therefore retained primarily at the study level. Formal funnel plots and Egger regression were not performed because the number of comparable estimates was insufficient.

Results

Search and Screening

The search produced 1249 records before date filtering. After removing seven records published after July 29, 2026, 1242 records remained. Thirty-eight duplicates were removed, leaving 1204 unique records for screening. Eight hundred ninety records were excluded during initial screening, and 314 reports were retained for detailed record-level eligibility assessment. Seventy-seven reports were subsequently excluded, leaving 237 reports that met the prespecified eligibility criteria at the record level and constituted the record-level eligible evidence base for this review.

Characteristics of the Identified Evidence Base

The PubMed results include studies and reviews addressing EEG, fNIRS, eye tracking, facial information, behavioral video, medical background information, neuroimaging, microbiome-related biomarkers, and other AI approaches. For example, one 2026 review identified 27 peer-reviewed EEG/fNIRS studies, while an eye-tracking systematic review identified 24 ML studies involving 1396 individuals [34].

Beyond direct ASD detection, related computational and engineering research has explored automated emotion labeling, wearable and digital interventions, facial-feature analysis, computer-vision measurement of autism-related behaviors, automated detection of stereotypical motor movements, and wearable biosensing for behavioral prediction [43-48].

Validation and Generalizability

The difference between development and independent validation is particularly important. Rajagopalan et al. reported an AUROC of 0.895 during development and 0.790 during independent validation [48]. Tariq et al. similarly reported lower performance under independent validation than in the held-out development environment [24]. These differences suggest that model performance may be sensitive to dataset composition and population characteristics.

Cross-cultural validation is particularly important for behavioral and video-based systems because social behavior, caregiver reporting, language, recording environments, and clinical referral pathways can vary across populations.

Representative Recent Evidence

Several records demonstrate the direction of the field.

Rajagopalan et al. developed an ML model using 28 medical and background variables from the SPARK cohort. Extreme gradient boosting (XGBoost) achieved an AUROC of 0.895 during development and 0.790 during independent validation [48]. Kim et al. evaluated an automated AI-based home-video approach among 510 children aged 18-48 months, reporting an AUC of 0.83 and accuracy of 0.75 [49]. This study is representative of the increasing movement toward low-burden, home-based behavioral assessment. Zhu et al. investigated multimodal response-to-name screening among toddlers, reporting an AUC of 0.81 and accuracy of 74.8% [50]. Salomon et al. examined real-world performance of the Canvas Dx AI-based diagnostic among children aged 18-72 months and reported sensitivity of 99.1% and specificity of 81.6% among determinate outputs [51].

These studies illustrate the increasing transition from retrospective algorithm development toward larger datasets, home-based assessment, and implementation-stage evaluation (Table 5, 6).

**Table 5:** Primary studies represented in the detailed study-level synthesis The table summarizes primary studies explicitly represented in the detailed study-level synthesis and does not represent the complete set of 237 record-level eligible reports. ASD - autism spectrum disorder; ADHD - attention-deficit/hyperactivity disorder; ML - machine learning; SRS - Social Responsiveness Scale; ABIDE - Autism Brain Imaging Data Exchange; fMRI - functional magnetic resonance imaging; LSTM - long short-term memory; XGBoost - extreme gradient boosting

Study	Data source/modality	AI/ML approach or focus	Role in ASD detection evidence
Wall et al., 2012 [11]	Behavioral diagnostic data	Artificial intelligence	Shortening behavioral ASD diagnosis
Wall et al., 2012 [12]	Observation-based behavioral data	Machine learning	Shortening observation-based ASD screening and diagnosis
Kosmicki et al., 2015 [13]	Behavioral features	Feature-selection ML	Identification of a minimal behavioral feature set for ASD detection
Duda et al., 2016 [14]	Behavioral/SRS features	Machine learning	Differentiation of ASD from ADHD
Duda et al., 2016 [15]	Mobile behavioral assessment	Mobile autism risk assessment	Clinical evaluation of mobile ASD risk assessment
Bone et al., 2015 [16]	Behavioral diagnostic data	Machine learning	Evaluation of ML approaches for facilitating ASD diagnosis
Abbas et al., 2018 [23]	Questionnaire and home video	Machine learning	Early ASD detection using combined questionnaire and video screening
Tariq et al., 2018 [24]	Home video	Machine learning	Development and prospective validation of mobile ASD detection
Tariq et al., 2019 [25]	Home video	Machine learning	Detection of developmental delay and ASD in Bangladeshi children
Washington et al., 2020 [26]	Behavioral video	Crowdsourced machine learning	Video-based recognition of autism-related behavioral features
Heinsfeld et al., 2018 [27]	ABIDE neuroimaging data	Deep learning	ASD identification using neuroimaging
Eslami et al., 2019 [28]	fMRI	Hybrid learning	ASD detection using ASD-DiagNet
Abraham et al., 2017 [29]	Multisite resting-state fMRI	Connectome-based classification	Multisite ASD classification and biomarker evaluation
Dvornek et al., 2017 [30]	Resting-state fMRI	LSTM	ASD identification using resting-state functional connectivity
Plitt et al., 2015 [31]	Functional-connectivity MRI	Classification modeling	ASD classification using functional-connectivity features
Nielsen et al., 2013 [32]	Multisite functional-connectivity MRI	Classification modeling	Multisite ASD classification using ABIDE data
Parisot et al., 2018 [33]	Neuroimaging	Graph convolutional network	Graph-based disease prediction including ASD
Rajagopalan et al., 2024 [49]	Medical/background information	XGBoost	ASD prediction using medical and background variables
Zhu et al., 2023 [51]	Response-to-name behavioral data	Multimodal machine learning	Early ASD screening among toddlers
Salomon et al., 2025 [52]	Clinical diagnostic data	AI-based diagnostic system	Real-world evaluation of AI-assisted ASD diagnosis

**Table 6:** Major data modalities and model families represented in the identified evidence ADI-R - Autism Diagnostic Interview-Revised; ADOS - Autism Diagnostic Observation Schedule; SRS - Social Responsiveness Scale; MARA - Mobile Autism Risk Assessment; SVM - support vector machine; XGBoost - extreme gradient boosting; CNN - convolutional neural network; LSTM - long short-term memory; EEG - electroencephalography; MEG - magnetoencephalography; MRI - magnetic resonance imaging; fMRI - functional magnetic resonance imaging

Modality	Examples of inputs	Representative models
Behavioral questionnaires	ADI-R, ADOS, SRS, MARA	SVM, logistic regression, decision trees, ensembles
Clinical/background information	Developmental and medical variables	XGBoost, logistic regression, random forests
Video/behavior	Home video, facial movement, response-to-name behavior	CNN, CNN-LSTM, multimodal models
Speech/acoustic data	Speech features, vocal characteristics	CNN, neural networks, transformers
Eye tracking	Gaze, fixation, scanpath characteristics	SVM, random forest, deep learning
EEG/MEG	Spectral power, coherence, connectivity	SVM, CNN, LSTM, graph models
MRI/fMRI	Structural and functional imaging	SVM, autoencoders, CNN, graph networks
Wearable sensors	Movement and behavioral signals	SVM, neural networks
Multimodal systems	Combined behavioral, clinical, video, or physiological information	Hybrid and ensemble architectures

Diagnostic Performance

Several behavioral and questionnaire-based models reported high classification performance.

Wall et al. reported accuracy approaching 100% in selected behavioral classification settings [11,12]. Kosmicki et al. reported accuracy above 97% for selected ADOS-based models [13]. Duda et al. reported an AUC of 0.965 for distinguishing ASD from ADHD using selected SRS features [14]. Tariq et al. reported an AUC of 0.933 on a held-out test set and 0.860 in independent validation [24]. Tariq et al.'s cross-cultural study reported AUC values of 0.76 and 0.85 in Bangladesh depending on the classification layer [25]. Neuroimaging studies generally reported lower study-level accuracy than the earliest behavioral models, with reported accuracy values commonly in the mid-60% to mid-70% range [27-33,36] (Table 7, Figure 3).

**Table 7:** Representative study-level diagnostic-performance estimates ADI-R - Autism Diagnostic Interview-Revised; ADOS - Autism Diagnostic Observation Schedule; SRS - Social Responsiveness Scale; MARA - Mobile Autism Risk Assessment; ADTree - alternating decision tree; SVM - support vector machine; SVC - support vector classifier; LDA - linear discriminant analysis; CNN - convolutional neural network; rs-fMRI - resting-state functional magnetic resonance imaging; MLP - multilayer perceptron; SLP - single-layer perceptron; L-SVM - linear support vector machine; L2LR - L2-regularized logistic regression; LSTM - long short-term memory; AUC - area under the receiver operating characteristic curve; NR - not reported

Study	Modality	Model	Accuracy	Sensitivity	Specificity	AUC
Wall et al. 2012 [11]	ADI-R	ADTree	99.90%	100%	98.70%	NR
Wall et al. 2012 [12]	ADOS	ADTree	100%	100%	94.40%	NR
Kosmicki et al. 2015 [13]	ADOS M2	Logistic regression	98.27%	98.81%	89.39%	NR
Kosmicki et al. 2015 [13]	ADOS M3	SVM	97.66%	97.71%	97.20%	NR
Duda et al. 2016a [14]	SRS	SVC/LDA	NR	NR	NR	0.965
Duda et al. 2016b [15]	MARA	ADTree	82.90%	89.90%	79.70%	NR
Tariq et al. 2018 [24]	Video + questionnaire	CNN + logistic regression	87.7% (held-out)	NR	NR	0.933 (held-out)
Tariq et al. 2018 [24]	Video + questionnaire	CNN + logistic regression	75.5% (independent)	NR	NR	0.860 (independent)
Tariq et al. 2019 [25]	Video + questionnaire	CNN + logistic regression	82%	75%	88%	0.76/0.85
Heinsfeld et al. 2018 [27]	rs-fMRI	Autoencoder + MLP	70%	74%	63%	NR
Eslami et al. 2019 [28]	rs-fMRI	Autoencoder + SLP	70.30%	68.30%	72.20%	NR
Plitt et al. 2015 [31]	rs-fMRI	L-SVM/L2LR	76.67%	70%	83.30%	NR
Abraham et al. 2016 [29]	rs-fMRI	Ridge/SVC	66.8% (inter-site)	61%	72.30%	NR
Dvornek et al. 2017 [30]	rs-fMRI	LSTM	68.50%	NR	NR	NR

**Figure 3:**
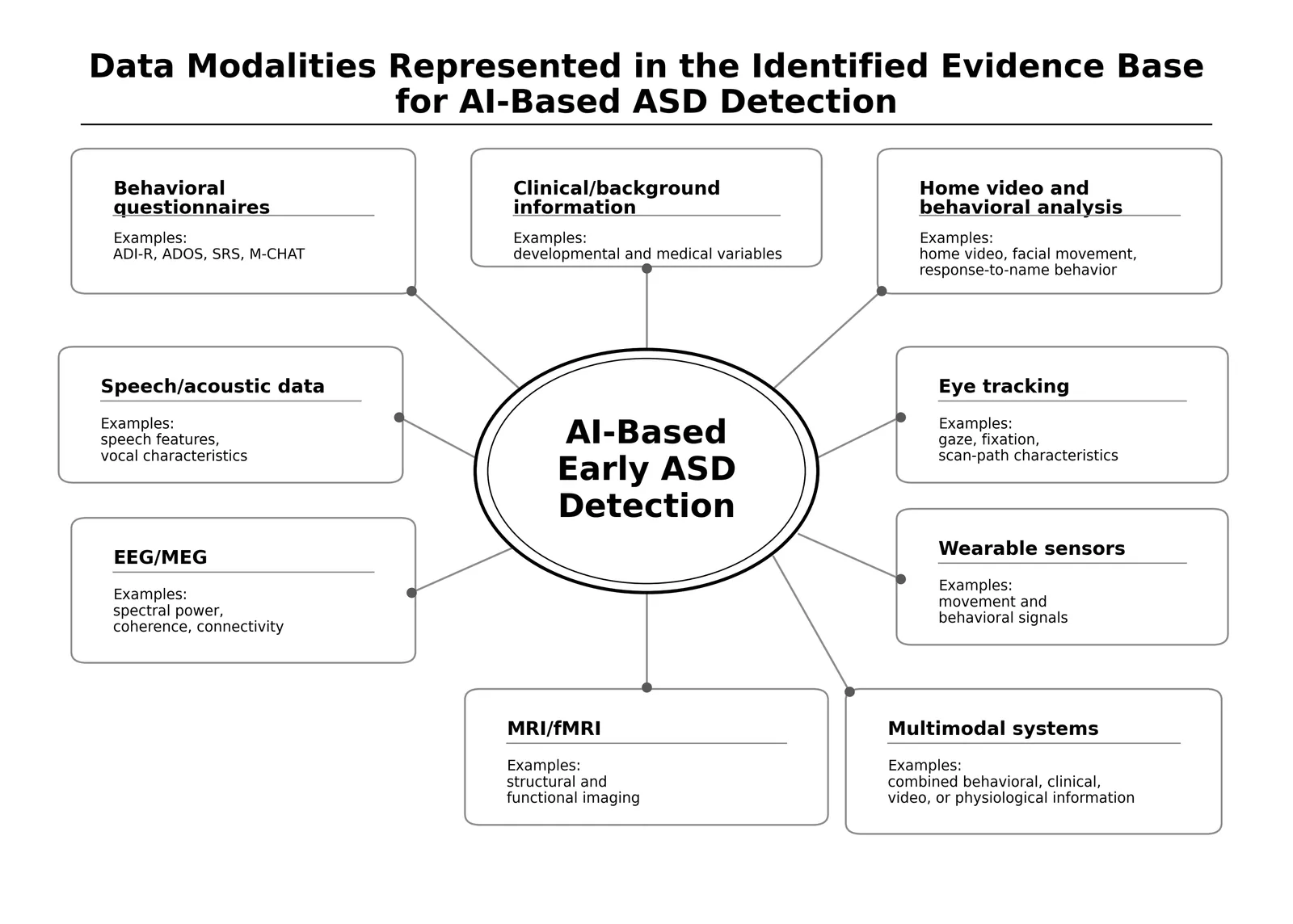
Major data modalities represented in the identified evidence base for AI-based ASD detection The figure summarizes the principal data modalities represented in the identified evidence base, including behavioral questionnaires, clinical/background information, home video and behavioral analysis, speech/acoustic data, eye tracking, wearable sensors, EEG/MEG, MRI/fMRI, and multimodal systems. Source: Created by the authors using Python (Matplotlib). ADI-R, Autism Diagnostic Interview-Revised; ADOS, Autism Diagnostic Observation Schedule; SRS, Social Responsiveness Scale; M-CHAT - Modified Checklist for Autism in Toddlers; ASD - autism spectrum disorder

Feature Engineering and Validation

Common feature-reduction strategies included principal component analysis, sparse or stepwise feature selection, mutual-information ranking, and recursive feature elimination [13,14,27-31]. Cross-validation was common, but prospective external validation was less consistently reported.

The strongest recurring methodological concerns were data leakage, dataset shift, class imbalance, inadequate external validation, limited demographic diversity, and insufficient reporting of preprocessing.

These concerns are important because accuracy may be inflated when preprocessing or feature selection occurs before proper separation of training and testing data.

Validation and Generalizability

The difference between development and independent validation is particularly important. Rajagopalan et al. reported an AUROC of 0.895 during development and 0.790 during independent validation [49]. Tariq et al. similarly reported lower performance under independent validation than in the held-out development environment [24]. These differences suggest that model performance may be sensitive to dataset composition and population characteristics.

Cross-cultural validation is particularly important for behavioral and video-based systems because social behavior, caregiver reporting, language, recording environments, and clinical referral pathways can vary across populations (Figure 4).

**Figure 4:**
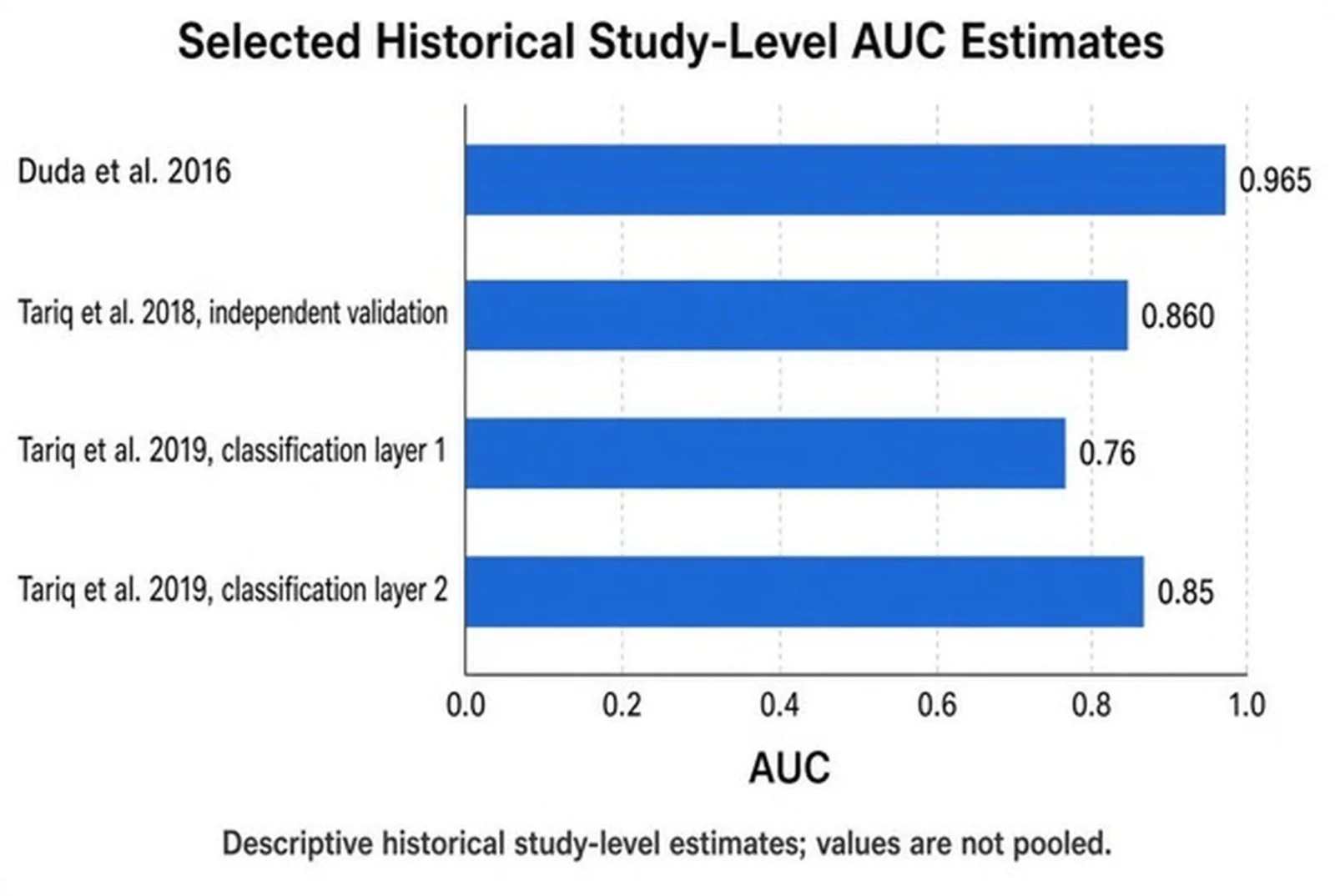
Selected study-level AUC estimates Sources: [14,24,25]. AUC - area under the receiver operating characteristic curve

Risk of Bias and Methodological Quality

Detailed PROBAST assessments were available for a subset of previously assessed primary studies for which full-text methodological information was available (Table 8). Complete full-text methodological information was not available for all newly identified records. Therefore, study-specific PROBAST+AI judgments were not extended to all 237 record-level eligible reports.

**Table 8:** PROBAST+AI reporting limitations identified from the available evidence Source: PROBAST and PROBAST+AI frameworks [41,42]. PROBAST+AI, Prediction Model Risk of Bias Assessment Tool + Artificial Intelligence; ASD, autism spectrum disorder.

Domain	Main concern
Participants	Representativeness and recruitment details often unavailable in abstracts
Predictors	Detailed preprocessing and feature-selection procedures often unavailable
Outcome	ASD classification was generally clear, but reference-standard details were often limited
Analysis	Leakage prevention, missing-data handling, calibration and tuning procedures often unavailable
Validation	External validation could not always be established from abstracts
Overall judgment	Definitive low/high risk was not assigned where information was insufficient

Table 9 shows engineering considerations for clinical translation and Table 10 shows the representative recent pediatric evidence identified in the current review.

**Table 9:** Engineering considerations for clinical translation Sources for the explainability considerations: [53,54] XAI - explainable artificial intelligence; DL - deep learning

Domain	Key concern	Recommended direction
Dataset quality and imbalance	Small or homogeneous samples and class imbalance can destabilize performance	Use diverse multisite datasets and class-aware metrics
Generalization and overfitting	Cross-validation alone may not establish transportability	Prioritize prospective and independent external validation
Data leakage	Preprocessing and feature selection can contaminate test data	Keep preprocessing and feature selection within training folds
Explainability	Opaque models may be difficult to audit	Evaluate clinically meaningful XAI approaches [53, 54]
Computational requirements	DL and imaging pipelines can require substantial resources	Evaluate efficient inference and model compression
Privacy	Pediatric behavioral and imaging data are highly sensitive	Use secure governance and privacy-preserving methods
Standardization	Acquisition and preprocessing vary across studies	Develop transparent standardized pipelines
Clinical deployment	Few systems are evaluated prospectively within workflow	Conduct prospective workflow and safety evaluation
Fairness	Models may underperform in underrepresented populations	Conduct demographic and cross- cultural validation

**Table 10:** Representative recent pediatric evidence identified in the current review AUC - area under the receiver operating characteristic curve; AUROC - area under the receiver operating characteristic curve; XGBoost - extreme gradient boosting; AI - artificial intelligence; SPARK - Simons Foundation Powering Autism Research for Knowledge

Study	Population	Modality/model	Validation	Key performance
Rajagopalan et al. 2024 [49]	SPARK cohort	28 medical/background features; XGBoost	Independent validation	Development AUROC 0.895; validation AUROC 0.790
Zhu et al. 2023 [51]	125 toddlers	Multimodal response-to-name behavioral analysis	Clinical cohort	AUC 0.81; accuracy 74.8%
Salomon et al. 2025 [52]	254 children aged 18-72 months	Canvas Dx AI diagnostic	Real-world clinical evaluation	Sensitivity 99.1%; specificity 81.6% among determinate outputs

Discussion

Principal Findings

The current search demonstrates substantial expansion in research applying ML and DL to ASD detection.

The literature is no longer concentrated primarily on questionnaire-based systems and neuroimaging. Contemporary research increasingly investigates home video, facial and behavioral analysis, speech, eye tracking, EEG/MEG, wearable sensors, clinical information, and multimodal combinations. This diversification may support development of lower-burden screening tools that can operate outside highly specialized clinical environments. However, expansion in the number of publications should not be interpreted as equivalent to evidence of clinical effectiveness.

Interpretation of Reported Performance

A significant finding is the variance in reported model performance. Reported accuracy depends on several methodological and dataset-related factors, including sample composition, class distribution, comparator selection, the diagnostic reference standard, preprocessing techniques, feature-selection techniques, model complexity, validation strategy, and dataset independence. Accordingly, an accuracy of 98% in one study in itself cannot certainly be interpreted as superior to an accuracy of 85% reported in another population because the underlying study designs, samples, and validation procedures may vary significantly. The importance of independent validation is demonstrated by Rajagopalan et al. [49], whose model attained an AUROC of 0.895 during development but decreased to 0.790 during independent validation. This reduction shows why performance estimates obtained during model development should not be presumed to represent performance in independent populations.

Behavioral and Questionnaire-Based Approaches

Questionnaire-based models remain attractive because questionnaires are relatively inexpensive and can be incorporated into existing developmental assessment pathways. However, their performance may depend on caregiver reporting, administration conditions, referral characteristics, and the population used for model development. The earliest behavioral studies reported extremely high classification accuracy [11-13], but such results should be interpreted alongside the validation strategy and comparator selection.

Video-Based and Remote Screening

Home-video approaches offer an attractive pathway for low-burden screening. Tariq et al. demonstrated that short home videos could be processed using ML to identify behavioral features associated with ASD [24-25]. Recent work continues this direction, including automated home-video analysis in children aged 18-48 months. However, home recordings introduce considerable variability in camera angle, lighting, audio quality, caregiver interaction, child engagement, and environmental context. Consequently, systems trained on standardized datasets may encounter substantial dataset shift in real-world deployment.

Neuroimaging and Physiological Approaches

Neuroimaging and electrophysiological approaches provide potentially objective biological information. MRI/fMRI studies have investigated functional connectivity and neuroanatomical patterns, while EEG and fNIRS studies have explored neural oscillations and hemodynamic responses. However, these approaches often require specialized equipment and technical expertise.

A 2026 review of EEG and fNIRS ML/DL studies found substantial heterogeneity in probe montages, preprocessing workflows, and classification models, limiting direct quantitative synthesis. The engineering challenge is therefore not simply to maximize classification performance but to develop reproducible pipelines that can operate reliably across acquisition systems and clinical environments.

Eye-Tracking Systems

Eye tracking represents another increasingly developed modality.

The evidence includes systems that analyze fixation patterns, gaze behavior, social attention, and scanpath characteristics. A systematic review and meta-analysis identified 24 eye-tracking ML studies involving 1396 individuals [34]. However, differences in devices, stimuli, calibration, preprocessing, participant engagement, and analysis strategies limit direct comparisons across studies.

Why High Accuracy Is Not Sufficient

The available evidence reinforces the principle that classification accuracy alone is insufficient to establish clinical usefulness. A model can achieve high accuracy due to class imbalance, highly selected case-control populations, data leakage, participant overlap between training and testing sets, inappropriate feature selection, test-set tuning, or other sources of optimism.

Clinically relevant systems should therefore report sensitivity, specificity, predictive values, calibration, confidence intervals where appropriate, and independent external validation.

PROBAST+AI and Methodological Quality

PROBAST+AI provides a framework for assessing prediction models developed using regression or AI methods [42].

Important considerations for ASD-AI research include participant selection, representativeness, predictor definition, outcome definition, sample size, missing data, feature selection, preprocessing, model development, model evaluation, validation, calibration, and applicability.

The abstract-only nature of much of the identified evidence means that several of these domains cannot be assessed confidently. This should be interpreted primarily as a limitation in available methodological information rather than automatically as evidence of high risk of bias.

Engineering and Clinical Translation

A clinically useful system requires appropriate data acquisition, secure preprocessing, reliable feature extraction, validated model inference, interpretable output, uncertainty estimation, appropriate referral thresholds, integration into clinical workflow, and computational efficiency.

A highly accurate model that requires excessive computational resources, specialized hardware, or lengthy inference may be unsuitable for low-resource or remote deployment. In lower-resource pediatric settings, questionnaire- and video-based approaches may be more feasible than imaging-based systems because they generally require less specialized equipment and infrastructure. Efficient architectures, model compression, edge inference, and deployment-aware design may therefore be important for future pediatric screening systems.

Ethical Considerations

Potential ethical concerns include algorithmic bias, privacy, informed consent, overdiagnosis, access inequities, transparency, responsibility for errors, and inappropriate automation [17-20,25,55].

Models trained primarily on narrow demographic or socioeconomic groups may not generalize equitably. Cross-cultural performance differences reinforce the importance of diverse validation populations. For pediatric applications, governance must also address the sensitivity and long-term implications of behavioral, video, speech, and neuroimaging data. AI-derived outputs should be communicated to families as probabilistic decision support rather than deterministic diagnosis.

Implications of the Evidence

The evidence identified in this review reinforces two major conclusions. First, clinically oriented ASD AI is increasingly being evaluated using lower-burden inputs such as response-to-name behavior, home video, questionnaires, and routinely available medical or developmental information rather than relying exclusively on resource-intensive neuroimaging. Second, AI systems are increasingly being evaluated in real-world or implementation-oriented settings.

This shift is important because a clinically useful system must perform not only under controlled research conditions but also within the variability of ordinary pediatric healthcare.

Comparison With Previous Reviews

The present review builds on previous behavioral, neuroimaging, eye-tracking, and broader AI reviews [21,22,34,36].

Earlier reviews already demonstrated substantial interest in ML for ASD detection. The present review shows that the research landscape has expanded in both modality and algorithmic complexity.

The contribution of the current review is therefore not the claim that AI-based ASD detection is a new research field. Instead, the contribution is the integration of multiple modalities with explicit consideration of engineering translation, validation, reproducibility, computational requirements, privacy, fairness, and clinical workflow.

Limitations

Several limitations should be acknowledged. First, the search was limited to PubMed and IEEE Xplore, and other databases may identify additional relevant evidence. Second, the database exports were available as bibliographic/abstract records, while complete full-text reports were not available for every candidate. Third, because of this limitation, the 237 record-level eligible reports should not be described as 237 full-text-confirmed included studies. Fourth, study-level PROBAST+AI assessment could not be completed for every record. Fifth, the evidence was highly heterogeneous in population, comparator group, modality, preprocessing, model architecture, and validation strategy. Sixth, only English-language publications were eligible, creating possible language bias. Seventh, some historically important studies used mixed-age datasets, and their findings should therefore not be interpreted as exclusively pediatric evidence. Eighth, the review was not prospectively registered in PROSPERO. Finally, the field is evolving rapidly, and continued surveillance of the literature will be important as new AI-based ASD detection systems emerge.

Directions for Future Research

Future research should prioritize prospective multicenter validation using independent and demographically diverse cohorts, incorporating blinded assessment and appropriate comparative designs where applicable, rigorous prevention of data leakage, participant-level separation of training and testing datasets, appropriate handling of class imbalance and missing data, transparent reporting of preprocessing and feature selection, calibration and uncertainty assessment, clinically meaningful explainability, privacy-preserving multi-institutional learning, evaluation of health equity and cross-cultural transportability, reproducible reporting and code availability, efficient deployment for mobile and low-resource settings, and prospective evaluation of AI systems within real clinical workflows.

Future Progress

Future progress depends less on incremental improvements in development-set accuracy and more on external validation, reproducibility, transparent methodology, explainability, privacy, fairness, calibration, computational efficiency, and integration into real pediatric clinical workflows (Figure 5).

**Figure 5:**
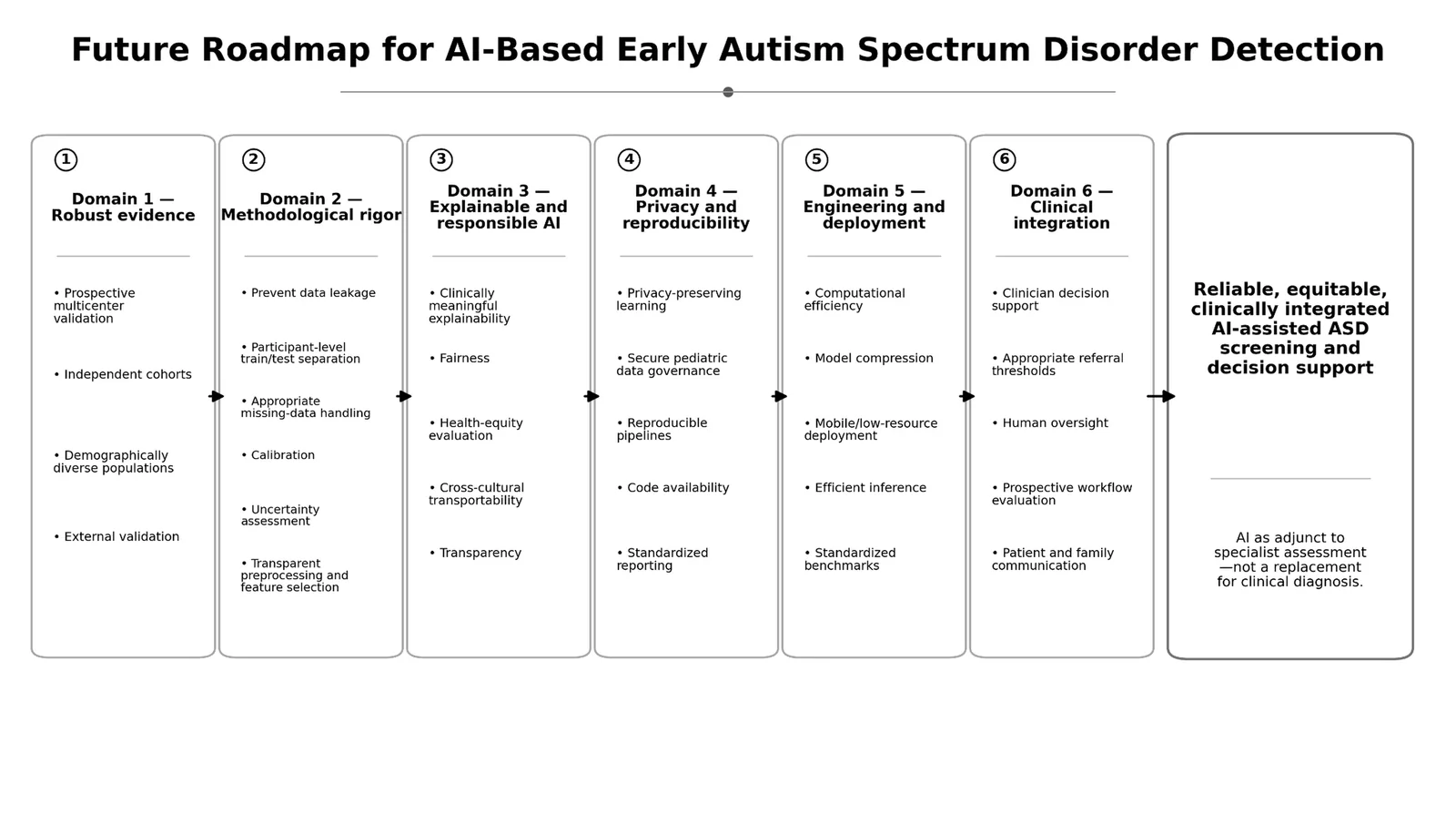
Future roadmap for AI-based early autism detection The figure summarizes key priorities for advancing AI-based early ASD detection toward clinical implementation, including robust evidence, methodological rigor, explainable and responsible AI, privacy and reproducibility, engineering and deployment, and clinical integration. AI is presented as an adjunct to specialist assessment rather than a replacement for clinical diagnosis. Source: Created by the authors using Python (Matplotlib). ASD - autism spectrum disorder; AI - artificial intelligence

## Conclusions

ML and DL approaches show considerable promise as adjunctive technologies for ASD screening and risk classification. The search demonstrates substantial expansion of the evidence base, including behavioral questionnaires, clinical information, home video, speech, eye tracking, EEG/MEG, MRI/fMRI, wearable sensors, and multimodal systems. Reported diagnostic performance varies substantially across studies and should not be compared without considering population, outcome definition, dataset, model-development strategy, and validation setting. Independent validation remains one of the most important requirements for translation. The evidence does not support replacing specialist ASD assessment with autonomous AI diagnosis. Instead, ML/DL systems should be developed as adjunctive decision-support, screening, or referral-prioritization tools, with appropriate human oversight.
